# Cell therapies and regenerative medicine - the dawn of a new age or more hype than hope?

**DOI:** 10.1186/2001-1326-1-12

**Published:** 2012-07-03

**Authors:** Anthony P Hollander

**Affiliations:** 1 School of Cellular & Molecular Medicine, University of Bristol, Medical Sciences Building, University Walk, Bristol, Clifton, BS8 1TD, UK

## 

Achieving success in the field of regenerative medicine will depend in part on our learning how best to manipulate cells *in vitro* in order to elicit therapeutic responses after implantation into patients. The traditional approach to tissue engineering is to combine cells with biomaterial scaffolds in order to create a template for tissue formation. There is a huge body of literature describing the science behind this type of approach but only relatively few clinical trials that shed light on the feasibility and efficacy of turning this interesting science into useful medicine [1]. A number of recent clinical studies have provided important insights into what might be achieved using a range of different techniques. Some examples of these are reviewed here in order to provide an overview of where this young field has got to and some of the challenges that lie ahead.

## State of the art in cell therapy

### Cell implantation without a supporting scaffold

The treatment of myocardial disease using intracoronary or intramyocardial bone marrow derived stem cells provides a fascinating insight into the challenges of using cell suspensions as drugs. Strauer and Steinhoff recently reviewed the first 10 years of this novel approach to the care of patients with heart disease [2]. They describe the first treatment in 2001 of a patient with a failing left ventricle, using unfractionated mononuclear bone marrow cells. In 2003 the first attempt was made to use a purified stem cell fraction [3]. This short report described the use of an anti-CD133 antibody to isolate bone marrow stem cells with potential angiogenic properties. This was a departure from the original concept of implanting cells that would restore contractility of the myocardium and instead focused on restoring the myocardial vascular supply. However it is becoming apparent that the same stem cells may both restore contractility (directly or indirectly) and improve the cardiac circulation [2]. Despite this lack of clarity on the proposed mechanism of action of stem cells, the extent of clinical improvement with these techniques is gradually becoming clear. Clinical results are highly variable and can be difficult to compare between studies because of huge variations in cell source, preparation and implantation methodology. Nevertheless there is merging evidence of a modest but significant improvement in ventricular performance in the treatment of acute myocardial infarction and increased ventricular performance and increased patient survival in the treatment of chronic infarction [2].

The rapid progression from laboratory to clinic in the treatment of myocardial disease has not been without controversy. In a 2004 Nature paper Murray et al provided evidence to oppose the idea that haematopoietic stem cells can transdifferentiate into cardiac myocytes [4]. Murray has publicly debated this issue with Sussman who is a proponent of the use of bone marrow cells for heart disease [5]. What is clear is that there is a need to understand more fully the mechanisms of action of these cell therapies so that improvements can be made through selection of cells with the greatest chance of contributing to the target mechanistic pathways. Iterative loops taking us from the laboratory, into the clinic and back again to provide increasingly refined cell therapies may be the only way to ensure that we develop effective and reliable treatments.

### Cell implantation on synthetic scaffolds

A bewildering array of biomaterials are being developed as potential scaffolds to combine with cells for tissue engineering [6]. Cells can be delivered on scaffolds to sites in the body requiring regeneration either as simple cell-scaffold constructs or after extensive pre-culture *in vitro* in order to generate a mature tissue engineered extracellular matrix prior to implantation [7]. Whilst the theory behind tissue engineering strategies favours a complete *in vitro* regeneration approach, the clinical reality is that investigators have favoured simpler methods involving delivery of cells within hours or days of seeding onto scaffolds. The tendency to avoid pre-matured tissues may come down to both the high cost of extended culture periods in a regulated laboratory, the difficulty of providing evidence of potency and purity when growing a complex tissue and the challenge of integrating pre-matured tissue with surrounding natural tissues at the implantation site. Example clinical studies are discussed here of cell delivery as a 2-dimensional patch for skin lesions and as a 3-dimensional implant for urethral reconstruction.

Moustafa et al have explored the feasibility of treating diabetic foot ulcers with autologous keratinocytes seeded onto a medical grade PVC dressing coated with acrylic acid by the technique of plasma polymerisation [8]. Their report illustrates some of the real difficulties in designing a clinical trial for tissue engineered products. Diabetic patients whose foot ulcers were refractory to conventional therapy were treated with 12 successive cell-scaffold constructs on a weekly basis. The control group were treated with cell-free dressings for 6 weeks and then converted to treatment with 12 cell-loaded scaffolds. Of the 16 patients originally recruited into the single-blind controlled trial, only 12 completed the study for a variety of reasons. There was tantalising evidence of efficacy, with 4 patients in the treatment group undergoing complete healing, but this effect failed to reach statistical significance due to the low numbers completing the study. Furthermore, some of the patients subsequently developed second reoccurrence of their ulcers, raising important questions as to the long-term viability of this approach.

However Raya-Rivera et al have demonstrated that meaningful data can be obtained even from an observational study of new cell therapies [9]. In this elegant study they treated 5 boys with traumatic urethral injuries with an engineered urethra. The implants were between 4 and 6 cm in length and were constructed from muscle and urothelial cells isolated from bladder biopsies and seeded onto tubularised polyglycolic acid scaffolds. By 3 months, normal urethra architecture had formed at the repair site. The boys were followed for up to 6 years after implantation and in all cases there was no need for further clinical intervention as they all remained continent.

Combination of cells and synthetic scaffold remain a viable option for developing relatively simple treatments for complex and intractable diseases.

### Cell implantation on decellularised natural tissues

An alternative to the use of cells combined with synthetic scaffolds is the combination of cells with decellularised organs. Under this approach, donated organs are decellularised in order to render the implant immune-compatible, avoiding the need for immunosuppressive therapy. The resulting complex natural scaffold is then repopulated with the patient’s own cells, thereby producing an autologous tissue engineered organ.

In 2008 we used this decellularisation and repopulation strategy to create the world’s first tissue engineered trachea [10,11], a 6 cm segment of the organ that was implanted into the left bronchus of a patient with severe bronchomalacia. The engineered organ was created from the patient’s own bone marrow derived mesenchymal stem cells that were expanded to a population of 6 million and then differentiated along the chondrogenic lineage before being seeded, in a bioreactor, onto a decellularised donor trachea from a cadaver. The patient’s own epithelial cells isolated from an upper airway biopsy were seeded onto the luminal surface to drive mucosal formation. The implant remains functional to date showing that by successfully replacing the donor cells with the patient’s we avoided the need for any immunosuppression.

The major question that now needs to be asked of this decellularisation approach is whether it can help us to overcome the problem of how to tissue engineer a complex organ. The trachea is a relatively simple tube with just two cell types. However the approach would be of most value in complex organ tissue engineering where synthetic scaffolds are unlikely to be able to provide a template for complete regeneration. Some fascinating studies are beginning to show the feasibility of complex organ regeneration. Ott et al have created bio-artificial rat hearts by decellularising a donor organ and repopulating it with a combination of endothelial cells and cardiac progenitor cells [12]. The hearts were maintained in bioreactors and tested for contractile and electrical activity. Some functional activity was recorded, albeit at a low level compared with natural hearts. In a directly analogous experiment, Soto-Gutierrez et al have decellularised rat liver and repopulated it with an expanded hepatocyte population [13]. The repopulated liver tissue showed good cell engraftment and good functional hepatic enzyme activity. We must be cautious not to over- interpret either the heart or liver tissue engineering studies as they both describe rat organs *in vitro*. The engineering of a human heart or liver would require a much larger cell number and so probably would be dependent on a good stem cell source. Furthermore there would need to be evidence of good efficacy *in vivo*. However despite these short-comings, the two studies are encouraging in showing that decellularisation can be used to produce an extracellular matrix template with the potential for cell engraftment and whole organ functionality.

## Future challenges

These scientific and clinical breakthroughs are exciting and show the real potential of cell therapies to cure diseases that were previously untreatable [1]. However there are several challenges we must overcome if we are to turn this new science into therapies that are in routine use around the world:

1. *Therapeutic profile.* Each therapy must have demonstrable efficacy and have minimal risk of side-effects. In this respect cell therapies are no different than drugs. However unlike pharmaceuticals, finding appropriate pre-clinical models in which to obtain robust data on efficacy and safety may sometimes be very difficult [14-16]. The first question will be which cell type to use for the animal model. If the intention is to use autologous human stem cells such as mesenchymal cells, should the pivotal animal studies be undertaken using human cells in immune compromised animals or animal cells that are the nearest equivalent to the human therapeutic? Similarly, if a human embryonic stem cell is to be used to generate an implant, should the *in vivo* studies use the same human stem cell lines or equivalent animal stem cells if they are available? A further difficulty is establishing a safe and effective dose range for the therapy. This will be easier for scaffold-free cells that are administered in suspension as cell number can be varied. Knowing how to adjust dose in a tissue engineering setting is more difficult. These issues are not insurmountable and indeed are being tackled by some research teams and biotechnology companies. However we have a long way to go to solve all of the issues.

2. *Potential for commercialisation*. The most effective way to make a cell therapy available to large numbers of patients will be to turn it into a commercial product that can be distributed world-wide [17]. This will require each therapy to be cost effective and simple enough a technology to scale up for mass-production [18]. For autologous therapies this will be particularly challenging as a profitable service-industry model will be required. These issues may seem profoundly un-academic, but if we fail to account for this need when developing our new techniques then the chances of their having a widespread impact will be much reduced.

3. *Regulatory issues*. The regulatory environment for cell therapies is becoming clearer [19,20] but remains very inconsistent between different territories. Similarly, there are worldwide variations in ethical and patenting approaches to cell therapies that will make successful exploitation of the emerging science particularly difficult. It is imperative that laboratory and clinical scientists are fully up to date with the rules and regulations for the different types of cell therapy in their own country and in each of those territories where they intend to provide access to the treatment.

## Hope or hype?

The exciting science and early clinical data provide us with every reason to be hopeful that our young science will bear fruit and that we will be able to treat many thousands of patients with cell therapies in the not too distant future. However we must also recognise the formidable hurdles that we must overcome if we are to be successful in our aims. These hurdles should not stop as from driving ahead with the translation of the laboratory techniques into therapeutics but we must underpin all our work with first rate science that will allow us to address the concerns about controlling efficacy and safety.In addition we must not ignore the commercial and regulatory issues that will be as important to resolve as the science. We are indeed on the threshold of a new age of therapeutics. We should be inspired by the possibility but remain realistic about how quickly we can satisfy the demands of patients for ever-better treatments.

## References

[B1] Trounson A New perspectives in human stem cell therapeutic research BMC Med 2009 7 29 10.1186/1741-7015-7-29 19519878 PMC2702289

[B2] Strauer BE Steinhoff G 10 years of intracoronary and intramyocardial bone marrow stem cell therapy of the heart: from the methodological origin to clinical practice J Am Coll Cardiol 2011 58 11 1095 1104 10.1016/j.jacc.2011.06.016 21884944

[B3] Stamm C Westphal B Kleine HD Petzsch M Kittner C Klinge H Autologous bone- marrow stem-cell transplantation for myocardial regeneration Lancet 2003 361 9351 45 46 10.1016/S0140-6736(03)12110-1 12517467

[B4] Murry CE Soonpaa MH Reinecke H Nakajima H Nakajima HO Rubart M Haematopoietic stem cells do not transdifferentiate into cardiac myocytes in myocardial infarcts Nature 2004 428 6983 664 668 10.1038/nature02446 15034593

[B5] Sussman MA Murry CE Bones of contention: marrow-derived cells in myocardial regeneration J Mol Cell Cardiol 2008 44 6 950 953 10.1016/j.yjmcc.2008.03.007 18440020 PMC2742337

[B6] Place ES Evans ND Stevens MM Complexity in biomaterials for tissue engineering Nat Mater 2009 8 6 457 470 10.1038/nmat2441 19458646

[B7] Farhadi J Fulco I Miot S Wirz D Haug M Dickinson SC Precultivation of engineered human nasal cartilage enhances the mechanical properties relevant for use in facial reconstructive surgery Ann Surg 2006 244 6 978 985 discussion 85 10.1097/01.sla.0000247057.16710.be 17122623 PMC1856618

[B8] Moustafa M Bullock AJ Creagh FM Heller S Jeffcoate W Game F Randomized, controlled, single-blind study on use of autologous keratinocytes on a transfer dressing to treat nonhealing diabetic ulcers Regen Med 2007 2 6 887 902 10.2217/17460751.2.6.887 18034628

[B9] Raya-Rivera A Esquiliano DR Yoo JJ Lopez-Bayghen E Soker S Atala A Tissue- engineered autologous urethras for patients who need reconstruction: an observational study Lancet 2011 377 9772 1175 1182 10.1016/S0140-6736(10)62354-9 21388673 PMC4005887

[B10] Macchiarini P Jungebluth P Go T Asnaghi MA Rees LE Cogan TA Clinical transplantation of a tissue-engineered airway Lancet 2008 372 9655 2023 2030 10.1016/S0140-6736(08)61598-6 19022496

[B11] Hollander A Macchiarini P Gordijn B Birchall M The first stem cell-based tissue- engineered organ replacement: implications for regenerative medicine and society Regen Med 2009 4 2 147 148 10.2217/17460751.4.2.147 19317632

[B12] Ott HC Matthiesen TS Goh SK Black LD Kren SM Netoff TI Perfusion-decellularized matrix: using nature's platform to engineer a bioartificial heart Nat Med 2008 14 2 213 221 10.1038/nm1684 18193059

[B13] Soto-Gutierrez A Zhang L Medberry C Fukumitsu K Faulk D Jiang H A whole-organ regenerative medicine approach for liver replacement Tissue Eng Part C Methods 2011 17 6 677 686 10.1089/ten.tec.2010.0698 21375407 PMC3103054

[B14] Gandolfi F Vanelli A Pennarossa G Rahaman M Acocella F Brevini TA Large animal models for cardiac stem cell therapies Theriogenology 2011 75 8 1416 1425 10.1016/j.theriogenology.2011.01.026 21463721

[B15] Regenberg A Mathews DJ Blass DM Bok H Coyle JT Duggan P The role of animal models in evaluating reasonable safety and efficacy for human trials of cell-based interventions for neurologic conditions J Cereb Blood Flow Metab 2009 29 1 1 9 10.1038/jcbfm.2008.98 18728679 PMC2682696

[B16] Muschler GF Raut VP Patterson TE Wenke JC Hollinger JO The design and use of animal models for translational research in bone tissue engineering and regenerative medicine Tissue Eng Part B Rev 2010 16 1 123 145 19891542 10.1089/ten.TEB.2009.0658

[B17] Mason C Manzotti E Regen: the industry responsible for cell-based therapies Regen Med 2009 4 6 783 785 10.2217/rme.09.72 19902995

[B18] Prescott C Polak J, Mantalaris S, Harding S The promise of stem cells: a venture capital perspective Advances in Tissue Engineering 2008 Imperial College Press, London 491 500

[B19] Munro C Harris N Polak J, Mantalaris S, Harding S UK regulatory issues: the view from the researcher Advances in Tissue Engineering 2008 Imperial College Press, London 537 557

[B20] Astori G Soncin S Lo Cicero V Siclari F Surder D Turchetto L Bone marrow derived stem cells in regenerative medicine as advanced therapy medicinal products Am J Transl Res 2010 2 3 285 295 20589167 PMC2892405

